# Examining the relationship between sport, physical activity and “Green Attitudes” among students in different university programmes

**DOI:** 10.3389/fspor.2025.1679891

**Published:** 2025-10-30

**Authors:** Marianna Moravecz, Eszter Centeri-Baraksó, Kinga Sikolya-Kertész, Karolina Eszter Kovács

**Affiliations:** ^1^ Institute of Physical Education and Sport Sciences, University of Nyíregyháza, Nyíregyháza, Hungary; ^2^ Institute of Mathematics and Informatics, University of Nyíregyház, Nyíregyháza, Hungary; ^3^ Department for Counselling, Developmental and School Psychology, Institute of Psychology, University of Debrecen, Debrecen, Hungary

**Keywords:** sport, environmentally conscious attitudes, spillover effect, Olympic factor, students

## Abstract

The United Nations 2030 Agenda framework highlights the role of sport in sustainable development in areas such as healthy lifestyles, well-being, quality education, gender equality and peaceful societies. Thus, the goals of Agenda 2030 are consistent with the goals of the Olympics. The IOC's holistic approach to the 2024 Paris Olympic Games went beyond environmental factors to include the sustainability of athletes' mental well-being. The main objective of our research is to map the role of green attitudes and sport in the sustainable habits of Generation Z university students aged 18–29 in the disadvantaged Northern Great Plain region. We examined three distinct fields of study: sports (*N* = 170), education (excluding physical education) (*N* = 176) and technical-agricultural (*N* = 170). The total number of respondents was 509 (*N* = 509). To collect data, an online questionnaire was used, which was completed voluntarily and anonymously. We sent the link to the questionnaire to students at the University of Nyíregyháza, thus ensuring that we only received responses from the region we wanted to study. The questionnaire was designed to collect information on six dimensions. The data were analysed using SPSS Statistics 25.0, in which we used Chi-square, Kruskal–Wallis test, Mann–Whitney test, and exploratory factor analysis. Concerning our first three hypotheses, based on the negative outlook of Generation Z. These young people face a declining social role and unfavorable economic conditions, which affect their general worldview, value system, and vision for the future. We point to the phenomenon of “neutral spillover”, which is an area where sport does not play a role in students' green environmental mindset. This is consistent with the previous findings, which indicate the uncertain living conditions of today's young university students. A novel aspect of our study is the Olympic factor developed in connection with the 2024 Paris Olympics and the “green” and “social” factors obtained within it. We use these to highlight the positive spillover effect of global sporting events, which influences university students' attitudes towards sustainability about mental well-being, providing an opportunity for sport to become a tool for sustainability education, especially among younger generations. Physical and mental well-being are crucial in the lives of the Generation Z surveyed, which reinforces the relevance of our topic regardless of field of study, gender and social status. As this generation has been socialised in the media world, the internet may also play a central role in terms of involvement in eco-conscious sporting events. This age group is referred to as the world's first global generation, the “Instant Online” generation, for whom multitasking is a defining lifestyle. Environmental challenges are becoming increasingly urgent, so it is essential to educate younger generations about the environment, even through international sporting events promoted by the media. Further research directions could include extending the survey to universities in different regions of the country and repeating the questionnaire longitudinally for comparison purposes.

## 1 Introduction

The relevance of our topic is highlighted by the isolation of athletes caused by the COVID situation, which was reinforced by the IOC in 2021 with the expansion of the Olympic motto for the Tokyo Olympics. The motto coined by Baron Pierre de Coubertin was expanded with the word “together”: “*Faster, higher, stronger—*together” (1). This word also symbolises people's concerns about our common sustainable future. According to recent research, those involved in sport accept and act on environmental goals (2, 3). Sport is deeply dependent on nature and closely linked to it; without a healthy planet, there is no arena for sport. The International Olympic Committee has joined the Sports for Nature initiative supported by the United Nations Environment Programme (UNEP). Launched in 2022, the initiative offers a roadmap for sports at all levels to become better stewards of nature. Why is this important? Extreme heat and air pollution, for example, affect athletes' health and disrupt competition schedules. Climate change is causing more extreme weather, such as floods that threaten sports facilities and other event-related infrastructure. Unfortunately, these impacts will only intensify, with even more tragic consequences for communities threatened by climate change, unless we act urgently.

According to sports ecology, sports movements are increasingly interacting with sustainable practices (4). Sport itself is presenting our society with new challenges. Thinking about a sustainable future came to fruition in the Paris Olympics, which can already be considered an eco-conscious Olympics in terms of its objectives. Efforts were made to minimise the construction of new facilities, solar and wind energy were used, plastic was banned from the competitions, and carbon neutrality was pursued. This was the first Olympics where, in addition to physical well-being, great emphasis was placed on the mental well-being of athletes.

During the event, several educational programs were launched to raise awareness of the importance of sustainability. Following a new holistic approach, they not only drew attention to environmental values, but also identified the sustainability of physical and mental well-being as a key area.

Athletes are under significant pressure, which can jeopardise their mental health. Approximately 35% of elite athletes suffer from some form of mental disorder, such as burnout, depression or anxiety (5). The IOC guidelines emphasise a holistic approach that includes prevention, support for well-being and encouraging dialogue on the importance of mental health.

The International Olympic Committee launched Athlete365 Mind Zone at the 2024 Paris Olympic Games, a first in Olympic history. A dedicated space was provided for athletes to relax and recharge, including virtual reality mindfulness experiences and activities such as postcard writing and painting, aimed at supporting the mental well-being of athletes before, during and after competitions. In this research, we focused specifically on sporting events, with a particular emphasis on the 2024 Paris Olympics. Overall, these Olympics provided an excellent example of how these good practices not only contributed to the success of the event, but also had a long-term positive impact on global sustainability efforts, inspiring others to implement similar initiatives.

The links between sport and sustainability are multifaceted (6). The United Nations has recognised sport as a relevant catalyst for sustainable development, recognising its power to raise awareness of climate protection and thereby promote greater community participation in local environmental issues. At the same time, sport has been identified as a tool for educating young people about environmental sustainability and climate change (7). As mentioned above, sport can have an impact in several areas. It promotes healthy lifestyles and general well-being, provides quality education, and improves concentration. It supports gender equality (8) and promotes peaceful social coexistence, and can contribute to reducing social inequalities (9). Sport itself is a unique opportunity at the global level that contributes in a specific way to human development. As a result, students of the new generation are already thinking more seriously about the future state of the environment and fundamental issues of sustainability (10). The UN's Sustainable Development Goals for 2030 include several targets that are indirectly related to the link between sport and environmental awareness. For example, when designing and operating sports facilities, they must be energy efficient, based on renewable energy sources and minimise their environmental impact.

Major sporting events, such as the Olympic Games or the FIFA World Cup (11, 12), have an environmental impact that, according to research, results in 10 million tons of greenhouse gas emissions annually, the vast majority of which originate from the Olympic Games and World Cups (13). Sports ecology now represents an extension of the rich scientific traditions of human ecology, covering several specific disciplines. This means that those involved in sports have a mutual impact on the natural environment. Sustainability is of paramount importance, as its results to date show that it can improve people's living conditions (14). In their research, Darnell and Millington (15) suggest that sport and “sport for development and peace” (SDP) should be positioned not as űa solution to the climate crisis, but as a fundamental aspect of the Earth's ecological life in the coming years (16).

It is necessary to thoroughly examine how sport influences the sustainability of young athletes' everyday habits, a phenomenon referred to in research as the “spillover effect” (17). This phenomenon means that attitudes and behaviours developed in the sporting environment can spill over into other areas of an individual's everyday life. In summary, the “spillover effect” is an economic, political or social phenomenon in which the effects of a given process or event extend beyond the original sector or region and affect another area (18). This effect is generally interpreted as a side effect of the original activity (19). This research points to the important role that sport plays in creating a culture of sustainability as a vehicle for positive values (20). Spillover effects can be positive, negative, or even neutral (21). Over the past 20 years, empirical research on spillover effects has made significant progress. It has been assumed that behavioural spillover can theoretically support people in their transition to a sustainable lifestyle (22, 23).

The investment appeal of the Olympic Games leads to local economic growth and job creation. In this case, social relations help strengthen links between the business and social spheres. We are not just talking about a sporting event, but also a cultural meeting place. Interactions between cultural diversity and international audiences strengthen global understanding and acceptance, thus promoting the educational role of openness and acceptance. The Games promote social cohesion and cooperation between nations, which can strengthen social ties in the long term. The development of these relationships can contribute to the development of individuals and communities (Table Annex 1). The values represented by Olympians, such as perseverance, struggle and team spirit, have an educational and formative role that can influence the personal development and future success of young people.

The organisers of the 2024 Olympic Games in Paris aimed to promote environmental, economic and social sustainability through sport and to set a new standard for future sporting events (24, 25). It is important to highlight sustainable infrastructure and facilities, as the organisers sought to maximise the use of existing facilities during the Games, thereby minimising the environmental footprint of new construction. Environmentally friendly building materials and energy-efficient technologies were used for the newly built venues. Renewable sources essentially covered the energy required for the event. Solar and wind energy were used, and energy-efficient systems were introduced to operate the facilities, reducing greenhouse gas emissions. The organisers' goal was to reduce waste production and maximise recycling. They introduced a ban on single-use plastics and encouraged the use of compostable or reusable materials in catering and hospitality. Another goal of the Paris Olympics was to create a carbon-neutral Olympics. Unavoidable emissions were offset by reforestation and other environmental projects, and climate awareness was promoted among the global community. During the event, several educational programmes were launched to raise awareness of the importance of sustainability. Following a new holistic approach, the focus was not only on environmental values but also on the sustainability of physical and mental well-being. Overall, these Olympics were a great example of how these good practices not only contributed to the success of the event but also have a long-term positive impact on global sustainability efforts, inspiring others to implement similar initiatives (Table Annex 2). The Paris Olympics (2024) sought to set a global example of sustainable development about sport, while reducing environmental impact, promoting physical and mental well-being and social justice.

The aim of our research is to examine the environmental attitudes and sustainable practices of university students living in the disadvantaged Northern Great Plain region. In addition, we would like to investigate whether there is a connection between sports and environmental attitudes. Our research questions are based on the study by Lenzi et al. (26), which we expanded to explore the relationship between global sporting events and university students' attitudes toward sustainability in connection with the 2024 Paris Olympics.

The research questions were divided to cover the areas to be examined as comprehensively as possible. Our main research question is: Is there a relationship between participation in sport (whether at the student or global level) and an environmentally oriented culture?

**H1:** We assume that the field of study is not a factor influencing the adoption of positive sustainable practices among students.

**H2:** We assume that students concerned about sustainability values will be overrepresented among students participating in teacher training.

**H3:** We assume that there will be no difference in the recognition of “green” issues between gender, social background, field of study, place of residence and physical activity.

**H4:** We assume that there will be no differences in terms of gender, social background, field of study, place of residence and physical activity about the two Olympic factors.

## 2 Material and methods

### 2.1 Sample

The research aims to examine the environmental attitudes and sustainable practices of students at different levels of education. Our research was conducted among Generation Z university students living in the disadvantaged North Great Plain region. We examined three distinct fields of study: sports, education (excluding physical education) and technical-agricultural studies. The total number of respondents was 509 (*N* = 509). Broken down by field of study, the students completed the questionnaire in almost equal proportions. There were 170 students studying sports, 176 studying education (but not physical education) and 163 studying technical and agricultural subjects. The processed data included 285 women and 224 men. In terms of age, we divided the respondents into four categories: the first group includes those aged 18–20, the second group includes those aged 21–23, the third group includes those aged 24–26, and the fourth group includes those aged 27–29. Among the respondents, those aged 18–20 (*N* = 191) and 21–23 (*N* = 162) are the most represented in university education. Those aged 24–26 (*N* = 47) are now very few, so we can conclude that this age group is actively working. Those aged 27–29 (*N* = 109) are continuing their studies. In terms of educational attainment, 82.3% of students have a high school diploma, 14.5% have a bachelor's degree, and only 3.1% have a master's degree. In terms of physical activity, the majority of respondents are active, with 65.6% engaging in physical activity and 34.4% not engaging in physical activity (Table 1).

**Table 1:** Socio-demographic characteristics of the sample (%) *N* = 509.

Variables	Characteristics
Gender	Male (44%)—Female (56%)
Education	High school diploma (82.3%)—Higher education (14.5%)—Master's degree (3.1%)
Social class	Lower class (12%)—Middle class (63.7%)—Upper class (24.4%)
Economic class	Barely adequate (18.1%)—Good (51.3%)—Very good (29.1%)—Poor (1.6%)
Employment	Employed (49.7%)—Unemployed (50.3%)
Type of settlement	Farm and village (18.5%)—Commune and small town (39.3%)—County seat (30.5%)—Capital city, large city (11.8%)
Physical activity	Physical activity (65.6%)—No activity (34.4%)
Type of sport	Team (30.3%)—Individual (28.7%)

### 2.2 Procedure

The research began at the end of August 2024 and ended in October. To collect data, we used an online Google Drive questionnaire, which was completed voluntarily and anonymously. We sent the link to the questionnaire to the students, and it took about 15 min to complete. The questionnaire was designed to collect information on six dimensions. The first dimension concerned the socio-demographic information of the sample, while the second dimension concerned sports and physical activity. The third dimension aimed to examine responses to environmental issues in order to understand students' attitudes and feelings towards this sensitive topic. The fourth dimension concerned the implementation of sustainable practices in physical activity and everyday life. The fifth dimension aimed to explore the respondents' value universe about the social and economic dimensions of sustainability. Finally, the sixth dimension covers sustainability issues related to the 2024 Paris Olympics from both a social and environmental perspective.

### 2.3 Statistical analysis

When formulating the research questions, the main consideration was to be able to make as accurate a comparison as possible with the results of Lenzi et al. (26). For this reason, in selecting statistical methods, where possible and permitted by the data, we endeavored to follow the methods used by Lenzi et al. (26).

To answer the research questions, we first summarised and analysed the data collected on the third, fourth, fifth and sixth dimensions. The third dimension was analysed using principal component analysis of the Likert scale, followed by the extraction of three factor indices (environmental alarm, environmental activism and environmental hostility). Based on the three factors obtained, we conducted further analyses of socio-demographic variables using Kruskal–Wallis and Mann–Whitney tests. The fourth dimension was analysed using an additive index (practical sustainability index). We examined the relationship between the practical sustainability index obtained from the fourth dimension and the students' fields of study by performing an independence test (Chi-square test). The average importance of environmental issues was analysed in the fifth dimension by comparing them with other social and economic issues on the scale. In the sixth dimension, similar to the third dimension, we obtained two factors (Olympic green factor, Olympic social factor) using principal component analysis of the Likert scale, which we used to perform comparisons using Kruskal–Wallis and Mann–Whitney tests for socio-demographic variables. The data obtained were analysed using the SPSS Statistics 25.0 software package, and in all cases, the non-parametric tests were preceded by a Kolmogorov–Smirnov test to check for normality.

An online survey was used for data collection, and due to the COVID-19 pandemic, the questionnaire was available online only. Data obtained were analysed using SPSS 25.0 software (27), including analysis of variance, two-sample *t*-test, Chi-square test and cross-tabulation analysis.

## 3 Results

More than 70% of respondents said that they always or often try to take most of these aspects into account in their daily lives (Table 2). More specifically, 77.1% of respondents said that they often or always try to reduce their energy consumption (lighting, air conditioning). In addition to energy consumption, they also pay special attention to water consumption, with 82% of students stating that they often or always try to reduce their water consumption. Similarly, 71.7% of respondents said that reducing plastic consumption was their preferred sustainable practice in their daily lives. In contrast, transport received little attention, with less than half of respondents (42.1%) saying they often or always avoid using it. This is the aspect that students pay the least attention to in their daily lives in terms of habits and sustainability. In addition, among sustainable practices, 63.3% of respondents answered “often” or “always” when asked about the sustainability of the products they use (clothing, accessories and appliances). Finally, 51.2% of respondents reported that they mostly use organic or plastic-free food products. The highest values were given to those practices that can be carried out in everyday life, while the lowest values were given to those practices that require time and money. This fact also stems from the socio-economic background of the disadvantaged region. The average of our index was 3.87, and the median was 4.

In the third dimension, similar to Lenzi et al. (26), we identified three factors that respondents considered equally important in terms of sustainability values. As a first step, we checked whether the conditions necessary for factor analysis (with varimax rotation) were met, and both the KMO value (0.906) and the Bartlett test (*p* < 0.001) confirmed that the variables were uncorrelated in pairs. The factor groups were created based on the factor weights of the factor matrix after rotation, which are shown in Table Annex 3. The values of the first factor can be summarised as those concerned about the environment (those who are concerned about the health of our planet), the second as environmentally indifferent (those who consider recycling useless), and the third factor as extreme activists (those who would participate in environmental protests if they could).

For questions related to the Paris Olympics, we again used a specific grouping method, factor analysis, which helped us create two Olympic factors. Following the procedure presented for sustainability values (KMO = 0.891; Bartlett's test *p* value < 0.001), we formed the new variables in this case as well based on the factor weights of the factor matrix after rotation, which are shown in Table Annex 4.

We named the two factors obtained as follows: (1) Olympic green factor, and (2) Olympic social factor. Looking at the composition of the two factors, it is striking that the issue of “ensuring physical and mental well-being” appears in both. This is because emotional, mental and physical well-being has become an almost daily necessity in the lives of Generation Z. The importance of this factor is also reflected in the key areas of the global objectives of the 2024 Paris Olympics (see Table Annex 2).

**Table 2:** Relative frequency of sustainable practices in everyday life.

Items	Never	Rarely	Often	Always	Often + always
I try to reduce energy consumption (lighting, air conditioning)	2.9%	20%	41.1%	36%	77.1%
I try to reduce water wastage	2.2%	15.9%	40.9%	41.1%	82%
I try to reduce my plastic consumption	2.9%	25.3%	37.5%	34.2%	71.7%
I try to use organic or plastic-free food products	12.4%	36.3%	31.6%	19.6%	51.2%
I make sure that the products I use (clothing, accessories, tools, etc.) are sustainable.	7.7%	29.1%	34.6%	28.7%	63.3%
I avoid means of transport, especially if they are for private use (e.g., car, scooter)	25%	33%	22.8%	19.3%	42.1%

After exploring and summarising the data structure, we will now examine the four hypotheses.

*In the first hypothesis (H1)*, we assume that the field of study is not a factor influencing the application of positive sustainable practices among students. Based on the results of the cross-tabulation (Chi-square = 11.273, *p* = 0.506), we accept our hypothesis. This is well supported by Cramer's association coefficient, which measures the strength of the relationship, with a value of C = 0.132 indicating a very weak relationship. We found the same result when we examined regular sports participation about the sustainability index.

*In the second hypothesis (H2)*, we assume that students concerned about sustainability values are overrepresented among students participating in teacher training. We used Kruskal–Wallis tests on the three variables obtained using factor analysis to test the second hypothesis. Based on the Kruskal–Wallis test value of *p* = 0.383, we reject the second hypothesis for those concerned about the environment**,** i.e., there is no dominance among the students' majors. The same cannot be said for the environmentally indifferent and extreme activist variables (environmentally indifferent factor: *p* = 0.033; environmental activists factor: *p* = 0.046). In the latter two cases, differences were found between students studying sports and technical sciences, which were well supported by the paired hypothesis tests.

*In the third hypothesis (H3)*, we assume that there is no difference in the recognition of “green” issues in terms of gender, social background, field of study, place of residence, and physical activity. We accept this hypothesis in all cases except for gender, which is well supported by the *p*-values obtained from the Kruskal–Wallis and Mann–Whitney tests in Table 3. Looking at Table 3, we can see that we reject the null hypothesis with 95% confidence in all cases for a single criterion, namely the gender of the students (since all *p*-values in this column are <0.05, and in this case, the group averages shown also clearly support their insignificance) (Table 3). Therefore, we can only partially accept the hypothesis.

**Table 3:** Significance values of the Kruskal–Wallis and Mann–Whitney tests based on demographic variables and green values.

Demographic variables	Field	Gender			Type of settlement	Social class	Physical activity
			Male	Female			
“Green values”	*p*-value	*p*-value	Average		*p*-value	*p*-value	*p*-value
Fire incidents	0.08	<0.001	7.04	8.11	0.280	0.065	0.389
Global warming	0.357	0.001	7.31	8.07	0.111	0.126	0.917
Deforestation	0.173	<0.001	7.16	8.18	0.291	0.085	0.787
Melting glaciers	0.118	<0.001	7.03	7.98	0.464	0.081	0.560
Environmental pollution	0.677	<0.001	7.17	8.1	0.391	0.065	0.772

In the *fourth hypothesis (H4*), we assume that there is no difference between the two Olympic factors in terms of gender, social background, field of study, place of residence and physical activity. This hypothesis can be considered a novelty in our research, as this study covers a topic that has not been researched before. We accept hypothesis H4 in all cases except for field of study and social background in the case of the Olympic green factor, which is well supported by the *p*-values obtained from the Kruskal–Wallis and Mann–Whitney tests.

Upon closer examination of the three cases in which the *p*-values obtained were below 5%, we found that sports students had a more positive opinion of the Olympic green factor than students from other fields. In terms of the Olympic social factor, engineering and agricultural students joined athletes in their positive assessment, while teacher training students underrated this issue. In terms of social class, the lower and middle classes share the same opinion and consider this year's Olympics less green than the upper social class. Thus, we can only partially accept the original hypothesis**.**

## 4 Discussion and conclusions

The background to the research was to investigate whether sport can be a tool for promoting sustainability and encouraging social change. The study aimed to explore the impact of sport on young people's environmental awareness and sustainable behaviour. We obtained a comprehensive picture of the sustainable habits and practices of students living in the disadvantaged North Great Plain region. We examined three distinct fields of study: sports, education and technical-agricultural studies. Based on the research of Lenzi et al. (26) and using factor analysis, we identified and examined three different conceptual forms: the environmentally concerned, the environmentally indifferent and the extreme activists. The spillover effect, which provides the theoretical background for our research, yielded interesting and unexpected results for the first three hypotheses of our research sample. Based on the negative future outlook of the researched Generation Z (28, 29), we point to the fascinating phenomenon of “neutral spillover,” an area where sport does not play a role in students' green environmental thinking.

Thus, we can answer our main research question, whether there is a relationship between participation in sport (either at the student or global level) and an environmentally oriented culture, as follows. Looking at our research sample, the results show that the group associated with the world of sport does not differ significantly from the rest of the sample in terms of sustainability issues. In this context, we encounter the phenomenon of “neutral spillover,” where the application of sustainability-oriented behaviors in the sports arena does not have a significant impact on daily habits. This result can be interpreted in light of the complex dynamics characteristic of spillover, which can manifest itself in positive, negative, or neutral forms (17, 21).

This is consistent with the findings of Lenzi et al. (26), which point to the uncertain living conditions of today's young university students. These young people face a declining social role and unfavourable economic conditions, which also affect their general worldview, value system and vision for the future. The Italian study (2023) focuses specifically on students' green attitudes and the role of sport, exploring how sport shapes their awareness and behaviour about sustainability. This research found that sport does not play a significant role in developing environmental awareness among students in Rome. Students tend to view sustainability in a broader sense and attribute only a minor role to sport, suggesting that further sports education interventions are needed to foster such attitudes.

Our research shows that major sporting events such as the Olympics have a significant negative impact on the environment, but that these adverse effects can be mitigated with appropriate measures. The 2024 Paris Olympic Games will not only focus on environmental factors but also the mental well-being of athletes. The IOC has introduced the most comprehensive mental health initiatives in the history of sport, including a programme to prevent online abuse and the Athlete365 Mind Zone. Our final hypothesis also draws attention to this factor. We can turn the neutral ripple effect into a positive one if we exploit the most characteristic traits of Generation Z, such as their susceptibility to media influence.

A novel aspect of our study is the Olympic factor developed in connection with the 2024 Paris Olympics and the “green” and “social” factors identified within it. The analysis of the Olympic factor revealed the positive impact of sport (world-class sporting events/Olympics) on university students' attitudes towards sustainability in terms of mental well-being. This ripple effect can already be interpreted as a positive “spillover” effect, which provides an opportunity for sport to become a tool for sustainability education and awareness-raising, especially among younger generations, who are paying increasing attention to environmental protection and sustainable lifestyles, both physically and mentally. Physical and mental well-being are key factors in the lives of the Generation Z sample, which reinforces the relevance of our topic regardless of profession, gender and social status. As this generation has been socialised in the media world, the internet may also play a central role in their involvement in eco-conscious sporting events.

The relevance of our research is highlighted by the new Olympic motto for 2021, “*Faster, higher, stronger—together*”, which was also reinforced by Erika Miklósa's thoughts on the topic of Sport and Sustainability at the Sport and Innovation Conference held at the Hungarian University of Physical Education and Sport on 17 October 2024. Her words echo the plans of Agenda 2030, which states that we can achieve a more peaceful society by promoting gender and national equality, including through sporting events. *“The 2021 Olympics placed a strong emphasis on thinking together, while the 2024 Olympics will focus on environmentally conscious thinking. Sports talents have a major role to play in conveying this message, as they are messengers and catalysts who can influence people. Sporting events such as the Olympics and sports personalities such as Olympians can influence the way ordinary people think about sport and sustainability.” (Erika Miklósa).*

Further research directions could include extending the survey to universities in different regions of the country and repeating the questionnaire longitudinally for comparison purposes. Our plans also include a detailed examination and comparison of the attitudes of individual and team athletes with regard to green attitudes.

## Data Availability

The raw data supporting the conclusions of this article will be made available by the authors based on individual request.

## Appendix

**Table ANNEX 1:** Spillover effect and social relations in terms of the capital elements concerned.

Type of capital	Spillover effect in relation to the 2024 Paris Olympics	Social relations and educational role
Economic capital	The Olympic Games attracted investment. They stimulated the local economy. They created new jobs.	New economic opportunities strengthened social integration. They boosted local entrepreneurship.
Cultural capital	Traditions and global participation have increased international cultural relations.	The events have promoted understanding, respect and acceptance of cultural diversity between different nations.
Social capital	The Olympics have helped to strengthen social ties at the global level, which may lead to long-term international cooperation.	Friendships and community ties between participating nations contribute to the development of young people's social skills and their integration into the global community.
Human capital	Athletes participating in the Olympics serve as role models, encouraging young people to participate in sports and contributing to the development of their skills and sense of responsibility.	The educational role of sporting achievements helps to foster perseverance and competitiveness in future generations, while strengthening the principles of cooperation and fair play.

Table compiled by the author based on (30).

**Table ANNEX 2:** Key areas for sustainability at the Olympics.

Key areas	Global goal	Direct goal
Key points of sustainability from the perspective of sporting events	Promoting sustainability through sports. Maximizing the use of renewable energy sources in the operation of sports facilities.	Presenting sustainable solutions. Setting transparent and credible examples for future sporting events. Covering at least 80% of the energy consumption of sports venues from renewable energy sources.
Sustainable products and consumption	Reducing the negative environmental impact of sports equipment and products. Minimize waste production and maximize recycling.	Use sustainable materials. Strengthen the local economy. Introduce selective waste collection at event venues. Promote waste-free food packaging.
Raising awareness of climate change and air pollution from sports games	Reducing carbon dioxide emissions. Optimizing the environmental impact of sports facilities. Reducing water consumption and using more efficient water management systems.	Making venues carbon neutral. Introducing water-saving technologies and rainwater harvesting systems in facilities.
Social and gender equality issues	Ensuring equal opportunities regardless of gender, social or cultural background.	Ensuring gender equality during the Olympic Games.
Diversity, physical and mental well-being, and broad community involvement	Promoting physical and mental well-being. Increasing community participation.	Actively involving different social groups in the organization and implementation of the event. Giving priority to local suppliers and partners in the organization of the event.

Olympics, 1st Sustainability and Legacy Report, 2024, edited by the author.

**Table ANNEX 3:** Factor matrix of sustainability values after rotation.

Items	Factors		
	Concerned about the environment	Indifferent to the environment	Esxtreme activists
The environmental crisis threatens our lives	.853		
Excessive consumption of raw materials is no longer sustainable for our planet	.832		
Pollution causes disease and death	.819		
Energy waste is an environmental problem	.812		
I am concerned about the health of our planet	.810		
Melting glaciers are destroying our future	.793		
The economic system will ruin our future	.574		
Keeping air conditioning on all the time is expensive and harmful to the environment	.475		
Global warming is a natural phenomenon, not caused by humans		.795	
Recycling collection is useless		.784	
Environmental pollution is a serious problem, but not as urgent as it is stated to be		.783	
What motivates me not to waste energy is primarily saving money		.769	
“It is pointless to sort my rubbish, it all gets thrown out anyway”		.758	
If I could, I would take part in environmental protests.			.826
Young people, who paint on the surface of works of art during environmental protests, are right			.809
I am angry at people who do not recycle			.684

**Table ANNEX 4:** Factor matrix after rotation for Olympic issues.

Items	Factors	
	Olympic green factor	Olympic social factor
The Olympics played a leading role in climate change and carbon dioxide emission reduction	.886	
In reducing local environmental air pollution	.866	
In promoting and consuming sustainable products	.731	
Ensuring physical and mental well-being	.578	.578
Accepting diversity		.875
Eliminating social inequalities		.757
In the broad involvement of the community		.737
